# Does Psychosocial Impact of Dental Aesthetics Questionnaire (PIDAQ) reflect the impact of malocclusion on facial aesthetics?

**DOI:** 10.1590/2177-6709.28.4.e232211.oar

**Published:** 2023-08-25

**Authors:** Mohammed FARAJ, Sundareswaran SHOBHA, Vadakkeypeediakkal LATHEEF, Prakash NIVEDITA

**Affiliations:** 1 Government Dental College, Department of Orthodontics & Dentofacial Orthopedics (Calicut, Kerala, India).

**Keywords:** PIDAQ, Quality of Life, Psychosocial impact, Facial Aesthetic Index, Malocclusion

## Abstract

**Introduction:**

Malocclusion is presumed to have adverse effects on dental and facial attractiveness, leading to deleterious psychosocial impact and Quality of life(QoL) of the individual. The Psychosocial Impact of Dental Aesthetic Questionnaire (PIDAQ) has proved to be effective for assessment of psychosocial impact of malocclusion on dental aesthetics, but it’s effectiveness for assessing facial aesthetics is unknown.

**Objective:**

The aim of the present study was to assess the effectiveness of PIDAQ on its ability to reflect the psychosocial impact of malocclusion on facial attractiveness, using the Facial Aesthetic index (FAI) after its translation and validation in the regional language.

**Methods:**

The 23-item PIDAQ, after translation process, cross-cultural adaptation and pilot testing, was administered to 330 subjects (62.5 % females and 37.5 % males; age range 18-30 years) with varying degrees of severity of malocclusion, assessed by the two components of the Index of Orthodontic Treatment Need (Dental Health Component, IOTN-DHC, for normative need; and self-administered Aesthetic Component, IOTN-AC, for subjective need) and FAI.

**Results:**

The internal consistency and test-retest reliability were good (Cronbach’s alpha = 0.859 - 0.958; Intraclass correlation coefficient =0.984). FAI, IOTN-DHC and IOTN-AC scores showed highly significant correlation with PIDAQ scores, depicting strong convergent validity (*p*< 0.001). One-way analysis of variance (ANOVA) and Bonferroni *post-hoc* test showed highly significant correlations (*p*-value < 0.001) for all comparisons. There were no significant differences between responses of males and females. The regional version of PIDAQ had excellent reliability.

**Conclusions:**

PIDAQ showed good psychometric properties and was able to effectively reflect the psychosocial impact of malocclusion on altered facial aesthetics.

## RESUMO

**Introdução:**

Acredita-se que a má oclusão tenha efeitos adversos na estética dentária e facial, levando a um impacto psicossocial e na qualidade de vida (QV) do indivíduo. O questionário PIDAQ (*Psychosocial Impact of Dental Aesthetic Questionnaire*) provou ser eficaz para avaliação do impacto psicossocial da má oclusão na estética dental, mas sua eficácia para avaliar a estética facial é desconhecida.

**Objetivo:**

O objetivo do presente estudo foi avaliar a eficácia do PIDAQ em refletir o impacto psicossocial da má oclusão na atratividade facial, usando o Índice de Estética Facial (FAI) após sua tradução e validação na linguagem regional.

**Métodos:**

O PIDAQ com 23 itens, após o processo de tradução, adaptação transcultural e teste piloto, foi administrado a 330 indivíduos (62,5% do sexo feminino e 37,5% do sexo masculino; faixa etária de 18 a 30 anos) com má oclusão de gravidade variada, avaliados pelos dois componentes do Índice de Necessidade de Tratamento Ortodôntico (Componente de Saúde Dentária, IOTN-DHC, para necessidade normativa; e Componente Estético auto-administrado, IOTN-AC, para necessidade subjetiva) e pelo FAI.

**Resultados:**

A consistência interna e a confiabilidade teste-reteste foram boas (alfa de Cronbach = 0,859-0,958; coeficiente de correlação intraclasse = 0,984). Os escores FAI, IOTN-DHC e IOTN-AC mostraram correlação altamente significativa com os escores do PIDAQ, representando forte validade convergente (*p*< 0.001). A análise de variância (ANOVA) de uma via e o teste *post-hoc* de Bonferroni mostraram correlações altamente significativas (*p* < 0,001) para todas as comparações. Não houve diferenças significativas entre as respostas de homens e mulheres. A versão regional do PIDAQ apresentou excelente confiabilidade.

**Conclusões:**

O PIDAQ mostrou boas propriedades psicométricas e foi capaz de refletir efetivamente o impacto psicossocial da má oclusão na estética facial alterada.

## INTRODUCTION

Physical characteristics are very important in the development of self-esteem and self-image.^1^ People with good facial aesthetics are invariably more confident and have higher self esteem.^1^ Malocclusion alters dentofacial aesthetics and influences a person’s body image, self confidence and even social integration.^2^ With facial aesthetics being such an important concern in today’s society, aesthetic improvement is often the most frequent subjective reason for seeking orthodontic treatment.^3^

Traditional methods of orthodontic assessment are invariably based on an objective evaluation of the face and occlusion, both clinically and cephalometrically.^4^ Patient’s self-perception of the malocclusion and how it affects his/her quality of life are usually not taken into consideration. The clinician’s assessment of the problem may not necessarily coincide with the patient’s reason for seeking orthodontic treatment, which in turn may lead to dissatisfaction from the patient’s viewpoint.^4^

Likewise, facial aesthetics may not be judged correctly by simply analyzing the occlusion. Sometimes facial profiles may be unacceptable, even in the absence of a malocclusion. Due to this, there is a paradigm shift in orthodontic diagnosis and treatment planning towards improvement of the soft tissues of the face. Goal-oriented approach specifies identification and preservation of positive attributes along with elimination of the negative ones.^5^

The need for an independent appraisal of the face has been previously recognized, and the Facial Aesthetic Index (FAI) was developed for this purpose in 2016.^6^ Recent studies on facial esthetic evaluation, the effect of an unfavourable inclination of the incisors on soft tissue aesthetics, the lack of acceptability of convex facial profiles induced by bimaxillary protrusion among black patients etc., have all referred to this index.^7^ ^-^ ^9^ However, the psychosocial impact of altered facial aesthetics has never been investigated so far.

Therefore it can be seen that malocclusion impacts both dental and facial aesthetics. To improve the overall quality of life, it is absolutely necessary to understand the psychosocial impact of malocclusion on both facial and dental aesthetics if the patient’s needs are to be considered adequately.^10^

There are many instruments assessing the oral health related quality of life (OHRQoL) primarily aimed at the adult population regarding caries, tooth loss, periodontal disease etc.^11^ ^,^ ^12^ Instruments measuring OHRQoL in children and adolescents like the Child Perception Questionnaire (CPQ^11^ ^-^ ^14^ ), Child-Oral Impact on Daily Performance (OIDP) etc., are not directly applicable to orthodontic treatment in young adults.^13^ ^,^ ^14^ One instrument that did address this issue was the Orthognathic Quality of Life Questionnaire, which was developed for use in patients requiring Orthognathic surgery.^15^ This is what lead to the development of the promising instrument specific to orthodontic aspects of OHRQoL, the Psychosocial Impact of Dental Aesthetics Questionnaire (PIDAQ) for young adults in the age range of 18-30 years, using a self-rated questionnaire.^16^

This questionnaire was initially developed in English as it was meant for the English speaking countries. Later on, it has been translated into many other languages after making appropriate adaptations, depending upon the social and cultural variations of the region.^17^ Brazilian, Chinese, Spanish, Nepalese, Italian, Moroccan Arabic, Turkish, Malayan English and Indian versions of the questionnaire have been published, demonstrating excellent validity and reliability.^17^ ^-^ ^25^ Obviously, PIDAQ effectively reflects patient perception of altered dental aesthetics. Malocclusion alters both dental and facial aesthetics. Whether PIDAQ would also be able to reflect the psychosocial impact of malocclusion-induced altered facial aesthetics has not yet been evaluated. From the clinician point of view, this would be a great advantage, as he/she would need to use only one instrument for assessing both issues. Hence it was decided to investigate this important aspect, for which PIDAQ needs to be translated and validated in the regional language.

Thus, after translation and cross-cultural adaptation to the regional language, the aim of this study was to:

» Assess the psychosocial effects of malocclusion-induced altered facial aesthetics, using PIDAQ.
» Assess the psychosocial effect of malocclusion, based on its severity and treatment need.
» Assess the effect of gender on the perceived Quality of Life (QoL) as affected by the malocclusion.

## MATERIAL AND METHODS

The PIDAQ is a psychometric instrument composed of 23 items. It has one positive and three negative domains. The four subscales are: Aesthetic Concern (AC), having 3 items; Psychosocial impact (PI), having 6 items; Social Impact (SI), having 8 items; and Dental self confidence (DSC), having 6 items. The possible responses for each item are marked using a 5-point Likert scale, as follows: zero = not at all; one = a little; two = somewhat; three = strongly; and four = very strongly. Later, occlusal irregularities and normative treatment need was assessed using the Dental Health Component (IOTN-DHC) and subjective need using the Aesthetic Component (IOTN-AC) of the Index of Orthodontic Treatment Need (IOTN).^26^

## TRANSLATION

The PIDAQ was first translated into the regional language, Malayalam (State of Kerala, India) by three orthodontic postgraduate students, who were proficient in both languages. They were familiar with QoL terminologies and instruments. Thus, version I of the questionnaire was formed.

## BACK TRANSLATION

The first draft of this version was translated back to English by a committee comprising of an English teacher and two dental postgraduate students. All three were proficient in both languages, but were unaware of the purpose of the study and had no knowledge of the original scale.

## ASSESSMENT OF TRANSLATIONAL QUALITY

The original and back translated versions of the questionnaire were compared by a committee comprising of two orthodontists and a general dentist, fluent in both languages, with good knowledge regarding QoL. The committee made recommendations, so that the back translated version would come as close to the original as possible.

## CULTURAL ADAPTATION

This was achieved with the help of another committee comprising of three orthodontists working in nearby hospitals and a community group in the district. They evaluated the questionnaire to see if the various concepts would be relevant to the cultural context of this society. Conceptual and semantic equivalence were assessed, and proposed modifications were made to improve accuracy and clarity. Thus an adapted Version II of the questionnaire was formed.

## PRETEST

**Figure 1:**
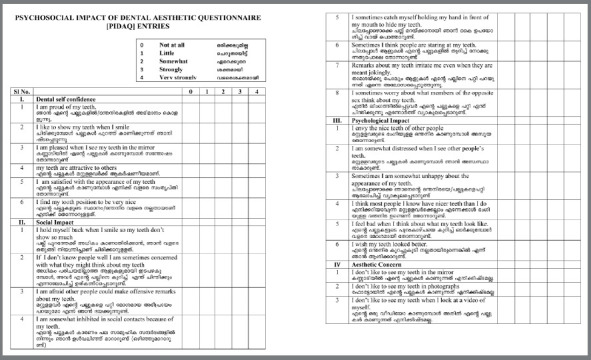
The adapted version of PIDAQ questionnaire.

This was followed by a pilot study conducted on a sample of 30 patients between the ages of 18-30 years comprising of 15 females and 15 males. The pilot test was performed by a single investigator (FM), through direct interview, to assess possible difficulties in understanding the questionnaire. At the end of this phase, appropriate adjustments were made and Version III of questionnaire was obtained (Fig 1).

The validity and reliability of the translated version of PIDAQ was carried out among the patients and students reporting for treatment at Government Dental College, Calicut, Kerala, India. Inclusion criteria were as follows: Only subjects who were willing to participate in the study, having full set of teeth, with no history of previous orthodontic treatment, no craniofacial anomalies, no carious / missing / fractured / fluorosed / discoloured teeth. 

Exclusion criteria were as follows: Students with physical disabilities preventing assessment of the questionnaire and mental / behavioural disorders that reduced their ability for self-determination. Sample size was determined to provide an 80% statistical power in identifying a significant difference in psychosocial impact, and was found to be 320. Later, 330 individuals in the age range of 18 to 30 years (mean 23 ± 0.6 years) were included in the study.

Approval for the study was obtained from the Institutional Research Committee and Institutional Ethics Committee of Government Dental College (IEC No: 112/2107/DCC). Informed consents from all the patients/students participating in the study were also obtained during the course of the research

The subjects were asked to fill up the PIDAQ questionnaire and were then examined by the trained orthodontist, without knowledge of their responses. Each patient took about 10-15 minutes to fill up the questionnaire. 

They were then examined for malocclusion using the Index of Orthodontic Treatment Need, based on Dental Health Component for normative need and Aesthetic Component for subjective assessment (IOTN, DHC & AC). Facial aesthetic assessment was based on FAI.

IOTN-AC - The patients were presented with ten photographs of the front teeth, displaying varying degrees of malocclusion, and were asked to indicate which grade of photograph (1-10) they think most closely resembled their own dentition. The ten IOTN-AC grades were combined into three groups: Grades 1-4 (slight), Grades 5-7 (moderate), and Grades 8-10 (definite).

The IOTN-DHC was assessed by the examiner and the various grades were combined into three groups: Grades 1-2 (no/slight malocclusion), Grade 3 (moderate) and Grades 4-5 (definite malocclusion).

**Figure 2:**
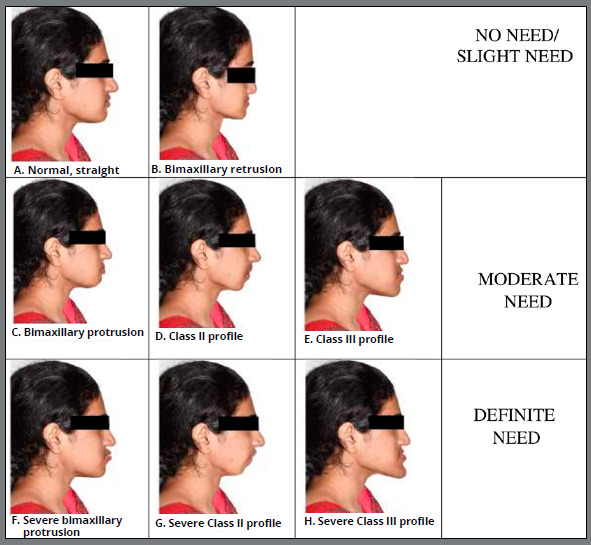
Representation of Facial Aesthetic Index chart, showing various profile variations.

**Figure 3:**
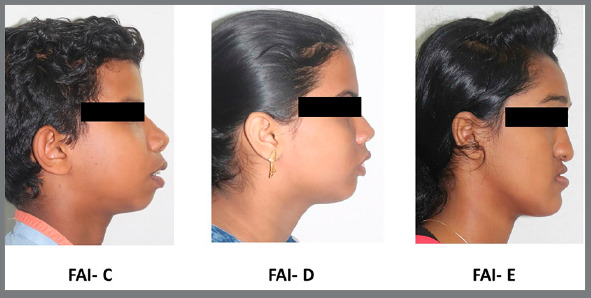
Representative photos of patients with moderate treatment need, based on Facial Aesthetic Index ( FAI ).

**Figure 4:**
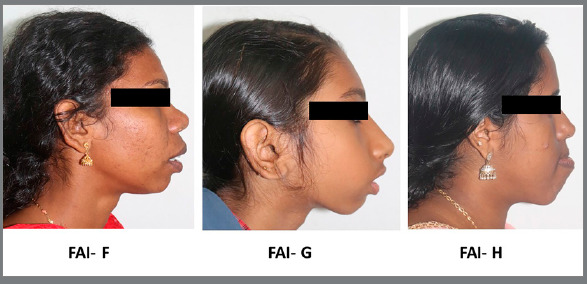
Representative photos of patients with more definite treatment need, based on Facial Aesthetic Index( FAI ).

FAI was assessed based on facial profile photographs depicting underlying malocclusions, which were coded from A to H (Table 1). In this investigation, FAI scoring A,B were grouped as “no need/ slight need”; C,D,E were grouped as “moderate need”; and F,G,H were grouped as “definite need”. The image depicting different facial aesthetic types is presented in Figure 2. Representative photographs of patients included in the study with moderate and more definite treatment needs, based on the Facial Aesthetic Index, are presented in Figures 3 and 4.

**Table 1:** The Facial Aesthetic Index description of various profiles and their codes.

Description	Profile	Code
Pleasing face, not needing orthodontic correction	Normal, Straight	A
Normal maxilla and mandible, with retrusive concave profile, prominent nose and chin. Upper and lower lips placed within the subnasale-pogonion line	Bimaxillary Retrusion	B
Normal maxilla and mandible, with protrusive dentoalveolar complex, leading to circumoral convexity, upper and lower lips positioned well ahead of subnasale-pogonion line, but competent	Bimaxillary Protrusion	C
Posteriorly divergent profile and a reduced profile angle (Glabella - Subnasale - Soft tissue pogonion <165^o^ ), competent lips	Class II profile	D
Anteriorly divergent profile and an increased profile angle >175º	Class III profile	E
Severely protrusive maxillary and mandibular dentoalveolar structures, with marked circumoral convexity, incompetent lips, very acute nasolabial angle, upper and lower lips positioned well ahead of the subnasale-pogonion line	Severe Bimaxillary Protrusion	F
Posteriorly divergent profile and a markedly reduced profile angle <165º, with inability to approximate the lips, due to normal maxilla with severely retrognathic mandible / severely prognathic maxilla with normal mandible, or combinations	Severe Class II profile	G
Anteriorly divergent profile and a markedly increased profile angle >175º, with inability to approximate the lips, due to normal maxilla and severely prognathic mandible / severely retrognathic maxilla and normal mandible, or combinations	Severe Class III profile	H

**Figure 5:**
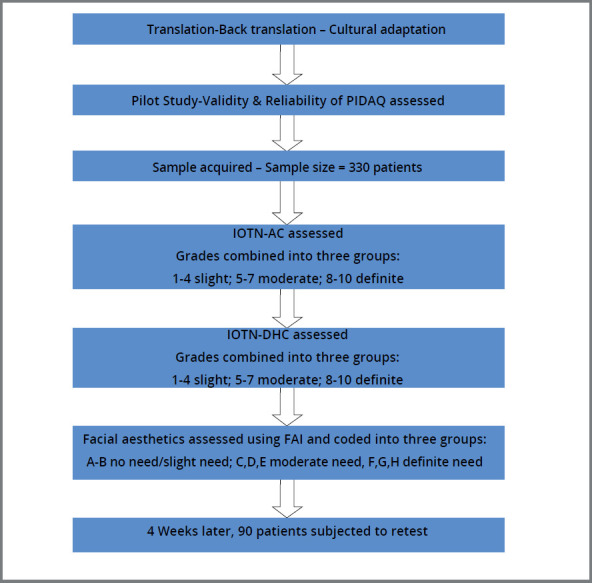
Flowchart depicting sequence of events in methodology.

After a period of four weeks, 90 subjects who reported again from among those contacted, were subjected to a retest to assess the reliability. A flowchart depicting the sequence of events is given in Figure 5. 

Convergent validity between PIDAQ score and IOTN-DHC, IOTN-AC and FAI was evaluated by ANOVA and Bonferroni *post-hoc* test. Construct validity of the tool was evaluated by Cronbach’s alpha.

## RESULTS

There was a total of 330 subjects, of which 37.5% were males. Within each domain and overall Qol, there were no statistically significant differences between males and females.

The internal consistency assessed by Cronbach’s alpha coefficient was 0.951 and it ranged from 0.859 (Psychological Impact, PI), 0.892 (Aesthetic Concern, AC), 0.898 (Social Impact, SI) and 0.918 (Dental self-confidence, DSC). The reproducibility assessed as test-retest reliability using intra-class correlation coefficient (ICC) was found to be 0.984 (p<0.001).

Table 2 shows the means and standard deviations and independent sample t-test of the responses obtained for all the different domains and overall QoL with respect to sex. Results showed that there were no significant differences between males and females with respect to all domains - DSC, SI, PI and AC. 

**Table 2:** Means and SD, with independent *t-*test of the responses obtained for all the different domains and overall Quality of Life (QoL), with respect to sex.

Variables		n		%	DSC		SI		PI		AC		Total
Sex	Age			Mean	SD	Mean	SD	Mean	SD	Mean	SD	Mean	SD
Male	18-30	124	37.5	23.09	6.20	18.41	8.58	16.44	6.44	9.40	3.98	67.34	22.28
Female	18-30	206	62.5	23.79	5.42	18.01	8.98	16.75	6.46	9.80	3.92	68.37	21.40
T value				-1.072		0.371		-0.415		-0.906		-0.419	
df				328		328		328		328		328	
P value				0.052		0.532		0.769		0.803		0.629	

n = Number of subjects. SD = Standard Deviation.

DSC = Dental self-confidence. SI = Social Impact. PI = Psychological Impact. AC = Aesthetic Concern.

* p < 0.05, ** p < 0.01, *** p < 0.001. QoL = Quality of life. T =*t*-test value. df = degree of freedom.

Table 3 shows the results of one-way analysis of variance (ANOVA) test comparing PIDAQ scores with different grades of malocclusion as categorized by IOTN-DHC, IOTN-AC & FAI rated by the subjects’ mean, standard deviations (SDs), F-statistics, and level of significance. 

**Table 3:** Results of one-way analysis of variance (ANOVA) comparing psychosocial impact of dental aesthetics questionnaire scores (Individual domains and Overall Sum) in respondents with different grades of malocclusion, as categorized by IOTN-DHC, IOTN-AC and FAI.

Domains	IOTN-AC			IOTN-DHC			FAI		
	S	M	D	S	M	D	S	M	D
DSC	22.04±5.79	27.29±2.35	28.00±2.94	20.85±5.89	24.96±4.22	27.35±3.48	20.20±6.60	25.12±4.06	29.38±0.80
F VALUE	37.49***			67.58***			46.276***		
SI	15.30±7.11	22.69±8.53	29.14±6.47	13.80 ±6.52	18.15±6.50	26.29±8.42	13.83±7.22	19.66±8.14	33.13±4.95
F VALUE	84.81***			155.96***			53.111***		
PI	14.60±5.61	20.51± 5.36	23.86±4.19	13.25±5.28	17.03±5.12	22.40±5.08	13.73±6.42	17.80±5.63	24.56±4.38
F VALUE	71.28***			144.22***			32.921***		
AC	8.64±3.77	12.26±2.76	12.78±2.87	7.98±3.68	10.15±3.49	12.34±3.07	7.45±4.03	10.62±3.22	14.56±0.96
F VALUE	38.75***			71.11***			47.786***		
SUM	60.61±18.6	82.74±15.4	93.78±13.0	55.96±17.8	70.30±15.5	88.37±15.9	55.21±21.2	73.21±17.1	101.63±9.2
F VALUE	87.98***			110.48***			63.058***		

*p < 0.05, **p < 0.01, ***p < 0.001. IOTN AC = Aesthetic Component of Index of Orthodontic Treatment Need. IOTN-DHC = Dental Health Component of Index of Orthodontic Treatment Need. FAI = Facial Aesthetic Index. DSC = Dental Self Confidence. S = Social Impact. PI = Psychological Impact. AC = Aesthetic Concern. S = SLIGHT. M = MODERATE. D = DEFINITE.

Table 4 depicts post hoc Bonferroni test to assess the relationship between domains of PIDAQ and different grades of malocclusion as evidenced by IOTN-DHC, IOTN-AC & FAI. The results reveal that the increasing PIDAQ scores reflect the impact of altered dental and facial aesthetics and the results were highly significant.

**Table 4:** Relationship between domains of PIDAQ and different grades of malocclusion as evidenced by IOTN-DHC, IOTN-AC & FAI ( Bon Ferroni post hoc test).

DOMAINS	GRADING OF SEVERITY		IOTN-DHC	IOTN-AC	FAI
DSC	SLIGHT	MODERATE	< 0.001***	< 0.001***	< 0.001***
		DEFINITE	< 0.001***	< 0.001***	< 0.001***
	MODERATE	DEFINITE	0.008**	1.000	0.004**
SI	SLIGHT	MODERATE	< 0.001***	< 0.001***	< 0.001***
		DEFINITE	< 0.001***	< 0.001***	< 0.001***
	MODERATE	DEFINITE	< 0.001***	< 0.001***	< 0.001***
PI	SLIGHT	MODERATE	< 0.001***	< 0.001***	< 0.001***
		DEFINITE	< 0.001***	< 0.001***	< 0.001***
	MODERATE	DEFINITE	< 0.001***	0.016*	< 0.001***
AC	SLIGHT	MODERATE	< 0.001***	< 0.001***	< 0.001***
		DEFINITE	< 0.001***	< 0.001***	< 0.001***
	MODERATE	DEFINITE	< 0.001***	1.000	< 0.001***
SUM	SLIGHT	MODERATE	< 0.001***	< 0.001***	< 0.001***
		DEFINITE	< 0.001***	< 0.001***	< 0.001***
	MODERATE	DEFINITE	< 0.001***	0.014*	< 0.001***

*p < 0.05, **p < 0.01, ***p < 0.001. Bonferroni comparison *post-hoc* test showing *p*-value < 0.001 for all comparisons, except for Moderate to Definite class of DSC and AC of IOTN-AC.

IOTN AC = Aesthetic Component of Index of Orthodontic Treatment Need. IOTN-DHC = Dental Health Component of Index of Orthodontic Treatment Need. FAI = Facial Aesthetic Index. DSC = Dental Self Confidence. SI = Social Impact. PI = Psychological Impact. AC = Aesthetic Concern.

## DISCUSSION

Structure of this version was similar to the original version of PIDAQ, as well as Brazilian and Spanish translations.^16^ ^-^ ^18^ The Chinese version, on the other hand, differs from the original in that it has only three domains, due to merging of Psychological Impact and Aesthetic Concern domains, to form one single domain called Aesthetic Attitude.^19^ Nepalese version has five domains, due to the addition of another domain termed Dental self-Consciousness.^20^

As severity of malocclusion increased, indicated by increase in scores of IOTN-DHC, there was definite increase in scores of DSC, SI, PI, AC and overall scores. This highly significant increase reflects the increasing concern of the subjects with respect to their occlusion and dental health. The statistically significant increase in the scores of the different domains, as the orthodontic treatment need progressed from slight need to moderate need and then to definite need, shows the ability of PIDAQ to discriminate between different levels of malocclusion.

Patient’s perception of malocclusion and subjective need of treatment is of profound importance. Professional evaluation of malocclusion may not always coincide with patient’s perception. The effect of self-reported degree of malocclusion on the QoL may be assessed with IOTN-AC. The present findings show that DSC, SI, PI and AC were highly affected by the IOTN-AC similar to the English, Italian and Chinese versions.^16,19,21^ Impact of increasing severity was reflected in the increasing DSC, SI, PI, AC and overall scores.

Patients seek treatment primarily for improvement of facial aesthetics. Evaluation of facial aesthetics is an important factor in overcoming psychosocial difficulties related to dental and facial appearance. Isolated correction of malocclusion should not be considered successful if facial aesthetics is not satisfactory at treatment completion.^27^ However, thus far, no attempts have been made to assess the psychosocial impact of malocclusion induced impaired facial aesthetics on the QoL of orthodontic patients. This is the first study making an attempt to assess this very important aspect. The QoL score of individuals was shown to deteriorate as the facial profile became worse, as assessed by FAI with respect to individual domains and overall QoL. An increasing trend in the scores was observed from no need /slight need, to moderate need, and to definite need treatment groups for DSC, SI ,PI, AC and overall QoL of the PIDAQ (p < 0.05). As the impact of facial profile aesthetics on OHRQoL has not been investigated so far, it was not possible to compare the present findings with previous research. This shows the ability of the current version of PIDAQ to differentiate the normal acceptable profile from other profile variations concomitant with different types and grades of malocclusion.

The present findings show that PIDAQ was able to reflect the impact of malocclusion effectively on both facial and dental aesthetics. Correlations representing convergent validity testing were strong between the PIDAQ domains and the self-assessed dental aesthetics, treatment need and facial profile aesthetics, as evaluated by ANOVA and Bonferroni *post-hoc* test, between PIDAQ score and IOTN-DHC, IOTN-AC and FAI. Construct validity was found to be good, as evidenced by Cronbach’s alpha. The reliability and reproducibility was also found to be excellent, as evidenced by the ICC scores.

There were no significant differences between males and females with respect to all domains (DSC, SI, PI and AC). This was similar to the Nepalese version.^20^ The high level of literacy and minimal difference between male and female literacy rates in this region probably accounts for the almost equal awareness among both males and females.^28^

The effect of transverse facial anomalies and asymmetries will not be reflected by the Facial Aesthetic Index. Hence psychosocial impact of transverse anomalies may not be detected. The sample in this study included undergraduate students with normal straight profiles also, with no need of orthodontic treatment. This facilitated correlation between normal subject that do not require orthodontic treatment with those that do need. Students from different parts of the country study in this Government institution; hence, the sample had adequate geographic representation of the target population.

## CONCLUSION

The following conclusions were drawn from this study:

1. The translated version of PIDAQ demonstrated excellent reliability and validity, with sufficient discriminative and evaluative psychometric properties.
2. PIDAQ was able to very effectively reflect the adverse psychosocial impact of malocclusion on facial aesthetics.
3. As the severity of malocclusion increased, leading to altered dental and facial aesthetics, a corresponding worsening of the quality of life (QoL) was observed, with emotional wellbeing/ psychological impact and social well-being domains of the individuals being the most affected.
4. There were no differences in QoL between the sexes, based on severity of malocclusion and facial profile aesthetics.
